# The quandary of anticoagulation for sepsis patients with new-onset atrial fibrillation

**DOI:** 10.62675/2965-2774.20250120

**Published:** 2025-02-28

**Authors:** Cassiano Teixeira, Túlio Frederico Tonietto

**Affiliations:** 1 Universidade Federal de Ciências da Saúde de Porto Alegre Department of Internal Medicine and Rehabilitation Sciences Porto Alegre RS Brazil Department of Internal Medicine and Rehabilitation Sciences, Universidade Federal de Ciências da Saúde de Porto Alegre - Porto Alegre (RS), Brazil.; 2 Hospital Nora Teixeira Intensive Care Unit Complexo Hospitalar da Santa Casa de Porto Alegre Porto Alegre RS Brazil Intensive Care Unit, Hospital Nora Teixeira, Complexo Hospitalar da Santa Casa de Porto Alegre - Porto Alegre (RS), Brazil.

Atrial fibrillation (AF) is the most common arrhythmia observed in clinical practice. New-onset AF is reported to occur in 2% to 44% of critically ill ICU patients, with the highest rates observed in patients with septic shock.^(1,2)^ A recent prospective study of 1,423 intensive care unit (ICU) patients revealed that 15.6% developed AF, with 13.3% of cases occurring during the ICU stay.^(3)^ AF triggered by secondary causes increases the risk of a cardiovascular event, particularly stroke, in the absence of anticoagulant therapy;^(3)^ however, the occurrence of new-onset AF does not significantly impact the risk of 90-day mortality.^(4)^

While anticoagulation reduces the risk of thromboembolic complications in outpatients with AF, its risk-benefit profile in critically ill patients with new-onset AF, both during and after intensive care unit (ICU) discharge, remains unclear owing to the lack of randomized clinical trials involving this population.^(3)^ Therefore, managing AF in patients with sepsis is challenging, given the competing risks of thromboembolism and bleeding. A recent systematic review highlighted two studies on anticoagulation: one revealed a 5% risk of bleeding with intravenous heparin, with no thromboembolic events, whereas the other revealed a 9% risk of bleeding with therapeutic anticoagulation, with no strokes reported during ICU admission.^(5)^ A systematic review by Nelson et al.^(6)^ revealed that AF patients receiving anticoagulants in critical care settings, including those with sepsis, had a higher incidence of major bleeding than those not receiving anticoagulants did, with no significant difference in the incidence of thromboembolic events. Walkey et al.^(7)^ investigated the use of oral anticoagulants after hospitalization due to sepsis with new-onset AF and reported that it was associated with a higher 1-year adjusted risk of ischemic stroke/TIA (5.69% *versus* 2.32%; risk difference, 3.37% [95%CI 0.36 - 6.38]), with no significant difference in major bleeding risk (6.51% *versus* 7.10%; risk difference, −0.59% [95%CI −3.09 - 1.91]). Moreover, long-term follow-up studies revealed that AF often recurred.^(1)^ The Framingham Heart Study^(8)^ revealed high recurrence rates due to secondary factors, with incidences of 42%, 56%, and 62% at 5, 10, and 15 years, respectively. In this population, the risks of stroke and mortality were not significantly different from those in individuals without secondary causes.

**Figure 1:**
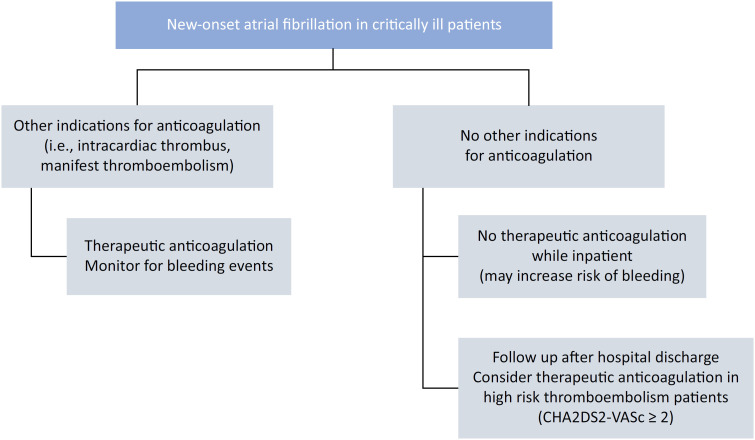
Flowchart proposed by the authors for anticoagulation therapy in critically ill patients with new-onset atrial fibrillation.

Given the inconsistent data on the benefits and harms of anticoagulant use among critically ill patients with new-onset AF, the high recurrence rate of AF after the initial episode, and the increased risk of post-ICU mortality from other causes (e.g., infections, falls), clinical decision-making can be challenging. Until more data are available, anticoagulation therapy for new-onset AF in the ICU appears to offer limited benefits and substantial risks. Anticoagulant therapy might not be worth the risk for these patients. After hospital discharge, owing to the increased risk of recurrent AF and its likely association with an increased risk of thromboembolic events, a personalized approach that includes vigilant monitoring for AF recurrence and the use of the CHA_2_DS_2_-VASc score may help guide therapy while further studies are underway. In summary, current evidence does not support routine anticoagulation therapy for AF during sepsis, as it increases the bleeding risk without a clear benefit in reducing the risk of thromboembolic events. After ICU discharge, clinical decisions should be tailored, and the risks and benefits for each patient should be carefully weighed (Figure 1).
